# Fast Finite-Time Observer-Based Event-Triggered Consensus Control for Uncertain Nonlinear Multiagent Systems with Full-State Constraints

**DOI:** 10.3390/e26070559

**Published:** 2024-06-29

**Authors:** Kewei Zhou, Xin Wang

**Affiliations:** 1 College of Westa, Southwest University, Chongqing 400715, China; swukeweizhou@163.com; 2 College of Electronic and Information Engineering, Southwest University, Chongqing 400715, China

**Keywords:** fast finite-time control, full-state constraint, event-triggered mechanism, unknown states and disturbance

## Abstract

This article studies a class of uncertain nonlinear multiagent systems (MASs) with state restrictions. RBFNNs, or radial basis function neural networks, are utilized to estimate the uncertainty of the system. To approximate the unknown states and disturbances, the state observer and disturbance observer are proposed to resolve those issues. Moreover, a fast finite-time consensus control technique is suggested in order to accomplish fast finite-time stability without going against the full-state requirements. It is demonstrated that every signal could be stable and boundless, and an event-triggered controller is considered for the saving of resources. Ultimately, the simulated example demonstrates the validity of the developed approach.

## 1. Introduction

The cooperative control of multiagent systems (MASs) has garnered more academic concerns in the last few decades [1,2], and meanwhile, it has been implemented in physical scenes including autonomous underwater vehicles and unmanned aerial vehicles [3,4]. Tracking consensus control is a particular case of cooperative control that has been used in multiple domains. Furthermore, the finite-time tracking consensus control method has been designed for heterogeneous MASs [5]. In [6], the authors used the asymptotic consensus control to achieve the asymptotic stability for MASs. The finite-time tracking consensus control approach is more effective than the asymptotic consensus control at preventing interference and converging more quickly. However, in physical situations, the system states and the external disturbances usually are unavailable but it is vital to achieve the consensus control of MASs [7,8].

In past research, there were various techniques to estimate unknown states and disturbances in the adaptive control field. A fuzzy adaptive output-feedback control strategy for uncertain switched nonlinear systems was put forth by Li and Tong [9]. The system was designed by the observer to solve the unmeasured states and unknown nonlinearities. Liu et al. [10] focus on adaptive fuzzy output feedback control for a category of nonlinear systems with unknown states and full-state restrictions. Otherwise, the disturbance observer had acquired remarkable achievement for MASs. In [11], Wang et al. built the disturbance observer to solve mismatched disturbance for the output consensus problem of leader–follower nonlinear MASs. Then, the event-triggered tracking control issue of nonlinear MASs with unmeasured disturbance was considered in [12]. Compared with the conventional research [9,10,11,12,13], the observer-based tracking consensus control for MASs with state restrictions has mostly not been investigated, which becomes a motivation for our research.

In addition, the traditional control scheme adopted time sampling to update the controller, which could cause a lot of resource waste in the practical industry. To do more with limited resources, the event-triggered mechanism would be proposed and receive widespread applications for MASs [14,15,16,17] due to its great effect on saving resources and reducing the number of triggers. In [15], Zhou et al. concentrated on event-triggered control problems for MASs with unavailable states, which also considered the tracking differentiator (TD) to deal with the uncertain nonlinear terms. The adaptive event-triggered control issue for uncertain nonlinear systems with full-state constraints and external disturbance was studied in [18] and TD was considered to deal with uncertain terms. However, they cannot be applied widely in MASs. Pang et al. [19] studied observer-based event-triggered control methods for MASs. Though Pang et al. considered unknown states and unknown disturbance in the event-triggered mechanism control strategy, the observer-based event-triggered consensus control problem for MASs with full-state restrictions has not been investigated.

Nevertheless, physical systems are subject to state restrictions for security reasons. Thus, full-state restrictions for system damage and performance deterioration are crucial to consider [20,21]. In [22], this research investigates an adaptive neural network control approach for stabilizing a type of uncertain strict-feedback system with full-state restrictions. Qu et al. [23] designed the state observer for nonlinear MASs with output constraints. Furthermore, Yao et al. [24] addressed a fixed-time consensus issue for nonlinear uncertain MASs with state restrictions. Although it has produced impressive results for MASs with state constraints, we discovered that no one has ever examined fast finite-time observer-based control for MASs with state restrictions, which is quite significant.

In traditional uncertain and nonlinear dynamic systems [22,23,24,25], they cannot consider the observer-based event-triggered control for MASs with full-state constraints; meanwhile, TD was first used to introduce controllers to solve such problems in MASS. This article considers the event-triggered consensus control scheme for MASs with full-state constraints. RBF NNs are designed to deal with uncertain and nonlinear terms. Meanwhile, the system has unmeasured states and external disturbances so that the state observer and the disturbance observer are designed. Additionally, to address the issue of “explosion of complexity”, the TD is considered.

The following are this paper’s main innovations:

1. The system states and external disturbance cannot be measured; the state observer and the disturbance observer enlarge the applications of the consensus control method.
2. A second-order TD is designed to avoid the continuous differentiations of the virtual control. Meanwhile, the event-triggered control approach can be investigated to save resources and reduce the frequency of the control triggering.
3. The MASs with state restrictions can achieve the stability and security of systems. In addition, the fast finite-time consensus control technique could make the system accomplish convergence at a high speed. In [26], the fast finite-time event-triggered control scheme do not consider the unmeasured states and disturbances, which also cannot have a good performance rather to our control method.

The remaining sections of the article are structured as follows. The dynamic and problem statement will be presented in Section 2. Section 3 and Section 4 propose the observer-based and event-triggered controller. In Section 5 and Section 6, the stability analysis and a simulation example are presented. Ultimately, conclusions are shown in Section 7.

**Assumption** **1.** *The reference signal and their derivatives are available and bounded.*

**Assumption** **2.** *For MASs(1), $|{\dot{f}}_{i,m}\left({\underline{x}}_{i,m}\right)|$, $|{\dot{d}}_{i,m}|$ and $|{\dot{u}}_{i}|$ satisfy that the positive constants $\kappa_{1},\kappa_{2},\kappa_{3}$ exist and we have $|{\dot{f}}_{i,m}\left({\underline{x}}_{i,m}\right)|<\kappa_{1}$, $|{\dot{d}}_{i,m}|<\kappa_{2}$ and $|{\dot{u}}_{i}|<\kappa_{3}$.*

## 2. Problem Statement and Dynamic

### 2.1. Graph Theory

The directed graph $G^{*}\left(V^{*},\varepsilon^{*},A^{*}\right)$ represent agents’ information in MASs, in which $V^{*}=\left\{v_{1},\ldots v_{N}\right\}$ and $\varepsilon^{*}=\left\{\left(v_{i},v_{j}\right)\in V\times V\right\}$ indicates the node set and the edge set, respectively. If $\left(v_{i},v_{j}\right)\in \varepsilon^{*}$, it indicates that one of Agent *i*’s neighbors is the follower *j*; meanwhile, the information can be sent to follower *i* by follower *j*. $N_{i}=\left\{j|\left(v_{i},v_{j}\right)\in \varepsilon \right\}$ expresses the neighbor set of the node *i*. The adjacency matrix of the graph $G^{*}$ is defined as $A^{*}=\left[a_{ij}\right]\in R^{N\times N}$ with $a_{ij}=1$, if $\left(v_{i},v_{j}\right)\in \varepsilon^{*}$; otherwise $a_{ij}=0$. Next, the Laplacian matrix for graph *G* is denoted by $L^{*}=D^{*}-A^{*}$, in which the degree matrix is $D^{*}=diag\left\{d_{1},\ldots ,d_{k}\right\}$ with $d_{i}=\sum_{j\in N_{i}}a_{ij}$.

### 2.2. System Definition

Consider the systems dynamics of follower *i* in MASs as

(1) $$\begin{array}{c} \left\{\begin{array}{c} {\dot{x}}_{i,m}=x_{i,m+1}+f_{i,m}\left({\underline{x}}_{i,m}\right)+d_{i,m}\left(t\right)\\ {\dot{x}}_{i,n_{i}}=u_{i}+f_{i,n_{i}}\left({\underline{x}}_{i,n_{i}}\right)+d_{i,n_{i}}\left(t\right),i=1,\ldots ,N\\ y_{i}=x_{i,1} \end{array}\right. \end{array}$$

where ${\underline{x}}_{i,m}={\left[x_{i,1},\ldots ,x_{i,m}\right]}^{T}$, and ${\underline{x}}_{i,n_{i}}=\left[x_{i,1},\ldots ,x_{i,n_{i}}\right]$; $f_{i}$ denotes unknown uncertain nonlinear functions. The system’s input is expressed by $u_{i}$, while its output is expressed by $y_{i}$. $d_{i}$ denotes the external disturbance. Additionally, owing to all state values being limited, it is defined by $|x_{i,m}|<\epsilon_{i,m}$ with $\epsilon_{i,m}>0$.

### 2.3. RBF NNs

In this article, because of their excellent performance, uncertain terms are estimated using radial basis function neural networks (RBF NNs). The basic formulation of RBF NNs is as follows:

(2) $$\begin{array}{c} \psi \left(X\right)=\theta^{*T}\Xi \left(X\right)+\Upsilon \left(X\right),X\in R \end{array}$$

in which $\Xi \left(X\right)={\left[\Xi_{1}\left(X\right),\ldots ,\Xi_{m}\left(X\right)\right]}^{T}$ denotes the basis vector function. $\theta^{*T}$ is the unknown constant vector. The approximation error Υ(X)≤r with r>0. Then, $\Xi_{i}\left(X\right)$ is the Gaussian function and has the following form:

(3) $$\begin{array}{c} \Xi_{i}\left(X\right)=exp\left[-\frac{{\left(X-\sigma_{i}\right)}^{T}\left(X-\sigma_{i}\right)}{c_{i}^{2}}\right],i=1,2,\ldots ,m \end{array}$$

where $\sigma_{i}={\left[\sigma_{i,1},\ldots ,\sigma_{i,n}\right]}^{T}$ and $c_{i}$ are the center and width of the basis function, respectively.

Consider ${\hat{\theta }}^{T}$ as the estimation of $\theta^{*T}$; then, the nonlinear uncertain function and the optimal parameter $\theta^{*}$ can be defined as follows:

(4) $$\begin{array}{cc} \hat{\psi }\left(\hat{X}\right)= & {\hat{\theta }}^{T}\Xi \left(\hat{X}\right) \end{array}$$

(5) $$\begin{array}{cc} \theta^{*}= & \arg\min_{x\in \Omega }\left\{\underset{x\in U}{\sup}\left|\hat{\psi }\left(\hat{X}\right)-\psi \left(X\right)\right|\right\} \end{array}$$

where the minimum fuzzy approximation error $\Delta =\hat{\psi }\left(\hat{X}\right)-\psi \left(X\right)$.

### 2.4. Preliminaries

**Lemma** **1**  ([26])**.** *For the system $\dot{x}=f\left(x\right)$, if the continuous function V(x), $g_{1}>0$, $g_{2}>0$, 0<ς<1, and 0<λ<∞ exist, the system is physically finite-time stable. Among them, $\dot{V}\left(x\right)$ satisfies $\dot{V}\left(x\right)\leq -g_{1}V\left(x\right)-g_{2}V^{\varsigma }\left(x\right)+\lambda$. Then the residual set of the system solution $\dot{x}=f\left(x\right)$ can be shown as follows:*

(6) $$\begin{array}{c} \lim_{x\to T_{e}}\leq \min\left\{\frac{\lambda }{g_{1}\left(1-\Lambda \right)},{\left(\frac{\lambda }{g_{2}\left(1-\Lambda \right)}\right)}^{\frac{1}{\varsigma }}\right\} \end{array}$$

*where* Λ *has 0<Λ<1, and the settling time $T_{e}$ are as follows:*

(7) $$\begin{array}{cc} T_{e}\leq \max\{t_{0}+ & \frac{1}{\Lambda g_{1}\left(1-\varsigma \right)}\ln\frac{\Lambda g_{1}V^{1-\varsigma }\left(t_{0}\right)+g_{2}}{g_{2}},\\ t_{0} & +\frac{1}{g_{1}\left(1-\varsigma \right)}\ln\frac{g_{1}V^{1-\varsigma }\left(t_{0}\right)+\Lambda g_{2}}{\Lambda g_{2}}\} \end{array}$$

**Lemma** **2**  ([27])**.** *For $z_{i}\in R$, i=1,…,N and o≤h≤1, one obtains*

(8) $$\begin{array}{c} {\left(\sum_{i=1}^{N}\left|z_{i}\right|\right)}^{h}\leq \sum_{i=1}^{N}{\left|z_{i}\right|}^{h} \end{array}$$

**Lemma** **3**  ([28])**.** *β is positive constant and 0<p<1.*

(9) $$\begin{array}{c} {\left(\frac{\alpha }{2\beta }\right)}^{p}\leq \frac{\alpha^{2}}{2\beta }+{\left(1-p\right)}^{\frac{p}{1-p}} \end{array}$$

### 2.5. Tracking Differentiator

Owing to the continuous differentiations existed in the virtual control, which could cause the problem of “Complexity explosion”, a second-order (TD) [29] is proposed with state variables $\chi_{i,m1}$ and $\chi_{i,m2}$. Furthermore, the input of TD is the virtual control $\alpha_{i,m}$. The estimations for $\alpha_{i,m}$ and ${\dot{\alpha }}_{i,m}$ are then displayed as follows:

(10) $$\begin{array}{c} \left\{\begin{array}{c} {\dot{\chi }}_{i,m1}=\chi_{i,m2}\\ {\dot{\chi }}_{i,m2}=-\delta *sign\left(\chi_{i,m1}-\alpha_{i,m}+\frac{\chi_{i,m2}\left|\chi_{i,m2}\right|}{2\delta }\right) \end{array}\right. \end{array}$$

where the acceleration factor δ>0. $\lim_{x\to \delta }\chi_{i,m1}=\alpha_{i,m}$ and $\lim_{x\to \delta }\chi_{i,m2}={\dot{\alpha }}_{i,m}$.

Then, the saturation function is utilized to replace the sign function to avoid unnecessary chattering near the origin. One obtains

(11) $$\begin{array}{c} \left\{\begin{array}{c} {\dot{\chi }}_{i,m1}=\chi_{i,m2}\\ {\dot{\chi }}_{i,m2}=-\delta *sat\left(\chi_{i,m1}-\alpha_{i,m}+\frac{\chi_{i,m2}\left|\chi_{i,m2}\right|}{2\delta }\right) \end{array}\right. \end{array}$$

where $sat\left(I.J\right)=\{\begin{array}{c} sign(I),|I|\geq J\\ \frac{I}{J},\left|I\right|\leq J \end{array}$ with J>0.

## 3. Observer and RBF NNs Adaptive Controller Design

According to Lemma 3 in [30], a state observer is proposed to solve the following unmeasured states:

(12) $$\begin{array}{c} \left\{\begin{array}{c} {\dot{\hat{x}}}_{i,m}={\hat{x}}_{i,m+1}+{\hat{f}}_{i,m}\left({\underline{\hat{x}}}_{i,m}|{\hat{\theta }}_{i,m}\right)+l_{i,m}\left(y_{i}-{\hat{x}}_{i,1}\right)+{\hat{d}}_{i,m}\left(t\right)\\ {\dot{\hat{x}}}_{i,n_{i}}=u_{i}+{\hat{f}}_{i,n_{i}}\left({\underline{\hat{x}}}_{i,n_{i}}|{\hat{\theta }}_{i,n_{i}}\right)+l_{i,n_{i}}\left(y_{i}-{\hat{x}}_{i,1}\right)+{\hat{d}}_{i,n_{i}}\left(t\right)\\ {\hat{y}}_{i}={\hat{x}}_{i,1} \end{array}\right. \end{array}$$

where ${\underline{\hat{x}}}_{i,m}={\left[{\hat{x}}_{i,1},\ldots ,{\hat{x}}_{i,m}\right]}^{T}$, and $\underline{\hat{x}}$ is the estimate of $\underline{x}$, $l_{i,m}$ is the observer gain. ${\hat{f}}_{i,m}\left({\underline{\hat{x}}}_{i,m}|{\hat{\theta }}_{i,m}\right)={\hat{\theta }}_{i,m}^{T}\Xi \left({\underline{\hat{x}}}_{i,m}\right)$ with ${\hat{\theta }}_{i,m}^{T}$ being the estimation of $\theta_{i,m}^{*T}$.

From Equations (4) and (5), one obtains

(13) $$\begin{array}{c} f_{i,m}\left({\underline{x}}_{i,m}\right)-{\hat{f}}_{i,m}\left({\underline{\hat{x}}}_{i,m}|{\hat{\theta }}_{i,m}\right)={\tilde{\theta }}_{i,m}^{T}\Xi \left({\underline{\hat{x}}}_{i,m}\right)+\Gamma_{i,m}+\Delta_{i,m} \end{array}$$

where ${\tilde{\theta }}_{i,m}=\theta_{i,m}^{*}-{\hat{\theta }}_{i,m}$, $\Gamma_{i,m}=\theta_{i,m}^{*T}\left(\Xi \left(\underline{x}\right)-\Xi \left(\underline{\hat{x}}\right)\right)$ and $e_{i}=x_{i}-{\hat{x}}_{i}$ is the observer error.

**Lemma** **4**  ([31])**.** *If $A_{i}$ being a strict Hurwitz matrix exists, for any matrix $N_{i}$, one obtains*

(14) $$\begin{array}{c} A_{i}^{T}M_{i}+M_{i}A_{i}=-N_{i} \end{array}$$

with $A_{i}=\left[\begin{array}{cccc} -l_{i,1} & & & \\ \vdots & & I_{i,n_{i}-1} & \\ -l_{i,n_{i}} & 0 & \ldots & 0 \end{array}\right]$.

To avoid the unknown external disturbance, the disturbance observer is intended to approximate $d_{i,m}\left(t\right)$; an auxiliary variable is suggested as follows:

(15) $$\begin{array}{c} z_{i,m}=d_{i,m}-\rho_{i,m}x_{i,m} \end{array}$$

where $\rho_{i,m}$ is a positive constant. According to (1), one obtains

(16) $$\begin{array}{c} {\dot{z}}_{i,m}={\dot{d}}_{i,m}-\rho_{i,m}\left(x_{i,m+1}+f_{i,m}\left({\underline{x}}_{i,m}\right)+z_{i,m}+\rho_{i,m}x_{i,m}\right) \end{array}$$

where $m=1,2,\ldots ,n_{i}$ and $x_{i,n_{i}+1}=u_{i}$.

Based on (14), we have ${\hat{z}}_{i,m}={\hat{d}}_{i,m}-\rho_{i,m}{\hat{x}}_{i,m}$. Next, ${\hat{z}}_{i,m}$ can be built as follows:

(17) $$\begin{array}{c} {\dot{\hat{z}}}_{i,m}=-\rho_{i,m}\left({\hat{x}}_{i,m+1}+{\hat{f}}_{i,m}\left({\underline{\hat{x}}}_{i,m}|{\hat{\theta }}_{i,m}\right)+{\hat{z}}_{i,m}+\rho_{i,m}{\hat{x}}_{i,m}\right) \end{array}$$

Combining (13), (15)–(17), ${\tilde{z}}_{i,m}$ can be built as follows:

(18) $$\begin{array}{cc} {\dot{\tilde{z}}}_{i,m}= & {\dot{d}}_{i,m}-\rho_{i,m}(e_{i,m+1}+{\tilde{\theta }}_{i,m}^{T}\Xi \left({\underline{\hat{x}}}_{i,m}\right)+\Gamma_{i,m}+\Delta_{i,m}\\ + & {\tilde{z}}_{i,m}+\rho_{i,m}e_{i,m}) \end{array}$$

where ${\tilde{z}}_{i,m}=z_{i,m}-{\hat{z}}_{i,m}$.

Consider the Lyapunov function $V_{e}$ as

(19) $$\begin{array}{c} V_{e}=e_{i}^{T}M_{i}e_{i}+\frac{1}{2}{\tilde{z}}_{i,m}^{2} \end{array}$$

where $M_{i}$ denotes a positive definite matrix. According to (1) and (12), one obtains

(20) $$\begin{array}{c} {\dot{e}}_{i}=A_{i}e_{i}+\sum_{m=1}^{n_{i}}\left(G_{i,m}{\tilde{\theta }}_{i,m}\Xi_{i,m}\left({\underline{\hat{x}}}_{i,m}\right)+{\tilde{d}}_{i,m}\left(t\right)\right)+{\bar{\Delta }}_{i} \end{array}$$

where $G_{i,m}={\left[0,\ldots m-2\ldots ,1,0,\ldots ,0\right]}^{T}$ and ${\bar{\Delta }}_{i}=\Gamma_{i}+\Delta_{i}={\left[\Gamma_{i,1}+\Delta_{i,1},\ldots ,\Gamma_{i,n_{i}}+\Delta_{i,n_{i}}\right]}^{T}$.

From Young’s inequality, we obtain

(21) $$\begin{array}{cc} 2e_{i}^{T}M_{i}\sum_{m=1}^{n_{i}}G_{i,m}{\tilde{\theta }}_{i,m}^{T}\Xi_{i,m}\left({\underline{\hat{x}}}_{i,m}\right)\leq & \frac{1}{2}{∥e_{i}∥}^{2}{∥M_{i}∥}_{F}^{2}+2\sum_{m=1}^{n_{i}}{∥{\tilde{\theta }}_{i,m}∥}^{2}\\ 2e_{i}^{T}M_{i}{\bar{\Delta }}_{i}\leq & \frac{1}{2}{∥e_{i}∥}^{2}{∥M_{i}∥}_{F}^{2}+2{\bar{\bar{\Delta }}}_{i}\\ 2e_{i}^{T}M_{i}\sum_{m=1}^{n_{i}}{\tilde{d}}_{i}\leq & n_{i}e_{i}^{T}e_{i}+{∥M_{i}∥}_{F}^{2}\sum_{m=1}^{n_{i}}{\tilde{z}}_{i,m}\\ + & \left(n_{i}+n_{i}{\bar{\rho }}_{i}\right)e_{i}^{T}M_{i}e_{i}\\ \sum_{m=1}^{n_{i}}{\tilde{z}}_{i,m}{\dot{d}}_{i,m}\leq & \frac{1}{2}\sum_{m=1}^{n_{i}}{\tilde{z}}_{i,m}^{2}+\frac{1}{2}n_{i}\kappa_{2}^{2}\\ \sum_{m=1}^{n_{i}}{\tilde{z}}_{i,m}\rho_{i,m}e_{i,m+1}\leq & \frac{1}{2}\sum_{m=1}^{n_{i}}{\tilde{z}}_{i,m}^{2}+\frac{1}{2}\left(n_{i}-1\right){\bar{\rho }}_{i}e_{i}^{T}e_{i}\\ -{\bar{\Delta }}_{i}\sum_{m=1}^{n_{i}}{\tilde{z}}_{i,m}\rho_{i,m}\leq & \frac{1}{2}\sum_{m=1}^{n_{i}}{\tilde{z}}_{i,m}^{2}+\frac{{\bar{\rho }}_{i}^{2}}{2}{\bar{\bar{\Delta }}}_{i}\\ -\sum_{m=1}^{n_{i}}{\tilde{z}}_{i,m}\rho_{i,m}^{2}e_{i,m}\leq & \frac{1}{2}\sum_{m=1}^{n_{i}}{\tilde{z}}_{i,m}^{2}+\frac{{\bar{\rho }}_{i}^{4}}{2}n_{i}{\bar{l}}_{i}e_{i}^{T}e_{i} \end{array}$$

where $∥{\bar{\Delta }}_{i}{∥}^{2}\leq {\bar{\bar{\Delta }}}_{i}$, ${\bar{\rho }}_{i}=max\left\{\rho_{i,m}\right\}$ and ${\bar{l}}_{i}=max\left\{l_{i,m}\right\}$. ∥•∥ denotes the Frobenius norm of a matrix.

Based on (18), (20), and (21), we obtain

(22) $$\begin{array}{cc} {\dot{V}}_{e}\leq - & e_{i}^{T}(\lambda_{min}\left(N_{i}\right)-\frac{1}{2}\left(n_{i}-1\right){\bar{\rho }}_{i}^{2}-\frac{1}{2}n_{i}{\bar{\rho }}_{i}^{4}-n_{i}\\ - & \left(n_{i}+n_{i}{\bar{\rho }}_{i}\right)∥M_{i}{∥}_{F}-∥M_{i}{∥}_{F}^{2})e_{i}\\ + & \sum_{m=1}^{n_{i}-1}(2-\rho_{i,m}+∥M_{i}{∥}_{F}^{2}){\tilde{z}}_{i,m}\\ + & (\frac{3}{2}-\rho_{i,n_{i}}+∥M_{i}{∥}_{F}^{2}){\tilde{z}}_{i,n_{i}}+\left(\frac{{\bar{\rho }}_{i}^{2}}{2}+2\right){\bar{\bar{\Delta }}}_{i}\\ + & \frac{1}{2}n_{i}\kappa_{2}^{2}-\sum_{m=1}^{n_{i}}\rho_{i,m}{\tilde{z}}_{i,m}{\tilde{\theta }}_{i,m}^{T}\Xi_{i,m}\left({\hat{\underline{x}}}_{i,m}\right) \end{array}$$

## 4. Adaptive Tracking Consensus Controllers Design

### Fast Finite-Time Event-Triggered Control Design

The consensus control approach can be proposed by the backstepping technique. Initially, we can define the synchronization error $\pi_{i,1}$ as follows:

(23) $$\begin{array}{cc} \pi_{i,1}= & \sum_{j\in N_{i}}\hbar_{ij}\left(y_{i}-y_{j}\right)+\tau_{i}\left(y_{i}-y_{0}\right) \end{array}$$

(24) $$\begin{array}{cc} \pi_{i,m}= & {\hat{x}}_{i,m}-\alpha_{i,m-1} \end{array}$$

where information transmits between the leader and the follower τ>0. Otherwise, τ=0. $\pi_{i,m}$ is the virtual control scheme. $y_{0}$ denotes reference signal.

*Step 1:* Based on [32], the barrier Lyapunov function will be selected as follows:

(25) $$\begin{array}{c} V_{i,1}=\frac{1}{2}log\frac{\epsilon_{i,1}^{2}}{\epsilon_{i,1}^{2}-\pi_{i,1}^{2}}+\frac{1}{2\mu_{i,1}}{\tilde{\nu }}_{i,1}^{2}+\frac{1}{2\eta_{i,1}}{\tilde{\theta }}_{i,1}^{2} \end{array}$$

where $|\pi_{i,1}|<\epsilon_{i,1}$, $\epsilon_{i,1}>0$, $\eta_{i,1}>0$ and ${\tilde{\nu }}_{i,1}=\nu_{i,1}-{\hat{\nu }}_{i,1}$.

Form (23), one yields

(26) $$\begin{array}{cc} {\dot{\pi }}_{i,1}= & c_{i}x_{i,2}+c_{i}f_{i,1}\left({\underline{x}}_{i,1}\right)+c_{i}d_{i,1}\\ - & \sum_{j\in N_{i}}\hbar_{ij}\left[x_{j,2}+f_{j,1}\left({\underline{x}}_{j,1}\right)+d_{j,1}\right]-\tau_{i}{\dot{y}}_{0} \end{array}$$

where $x_{i,2}=e_{i,2}+\pi_{i,2}+\alpha_{i,1}$ and $c_{i}=\sum_{j\in N_{i}}\hbar_{ij}+\tau_{i}$.

Adopt RBF NNs to address the unknown uncertain function as follows:

(27) $$\begin{array}{cc} F_{i,1}\left({ℜ}_{i,1}\right)= & c_{i}\left(e_{i,2}+\pi_{i,2}+f_{i,1}\left({\underline{x}}_{i,1}\right)+d_{i,1}\right)\\ - & \sum_{j\in N_{i}}\hbar_{ij}\left[x_{j,2}+f_{j,1}\left({\underline{x}}_{j,1}\right)+d_{j,1}\right]-\tau_{i}{\dot{y}}_{0}\\ = & \varpi_{i,1}^{T}\Psi_{i,1}\left({ℜ}_{i,1}\right)+\Phi_{i,1}\left({ℜ}_{i,1}\right) \end{array}$$

where ${ℜ}_{i,1}={\left[{\underline{x}}_{i,1},{\underline{x}}_{j,2},d_{i,1},d_{j,1},y_{0},{\dot{y}}_{0}\right]}^{T}$.

Next, based on Young’s inequality, we obtain

(28) $$\begin{array}{cc} \varpi_{i,1}^{T}\Psi_{i,1}\left({ℜ}_{i,1}\right){\bar{\pi }}_{i,1}\leq & \frac{{\bar{\pi }}_{i,1}^{2}\nu_{i,1}\Psi_{i,1}^{T}\Psi_{i,1}}{2m_{i,1}^{2}}+\frac{m_{i,1}^{2}}{2}\\ \Phi_{i,1}{\bar{\pi }}_{i,1}\leq & \frac{{\bar{\pi }}_{i,1}^{2}}{2}+\frac{{\bar{\Phi }}_{i,1}^{2}}{2} \end{array}$$

where $\nu_{i,1}={∥\varpi_{i,1}∥}^{2}$, ${\bar{\pi }}_{i,1}=\frac{\pi_{i,1}}{\epsilon_{i,1}^{2}-\pi_{i,1}^{2}}$ and $m_{i,1}>0$.

Next, we can consider the virtual control scheme $\alpha_{i,1}$ and the adaptive laws ${\dot{\hat{\nu }}}_{i,1}$ and ${\dot{\hat{\theta }}}_{i,1}$.

(29) $$\begin{array}{cc} \alpha_{i,1}= & -\frac{1}{c_{i}}\left[a_{i,1}\pi_{i,1}+\frac{b_{i,1}\pi_{i,1}^{2p-1}}{{\left(\epsilon_{i,1}^{2}-\pi_{i,1}^{2}\right)}^{p-1}}+\frac{{\bar{\pi }}_{i,1}^{2}}{2}+\frac{{\bar{\pi }}_{i,1}\nu_{i,1}\Psi_{i,1}^{T}\Psi_{i,1}}{2m_{i,1}^{2}}\right] \end{array}$$

(30) $$\begin{array}{cc} {\dot{\hat{\nu }}}_{i,1}= & \frac{{\bar{\pi }}_{i,1}^{2}\Psi_{i,1}^{T}\Psi_{i,1}\mu_{i,1}}{2m_{i,1}^{2}}-2g_{i,1}{\hat{\nu }}_{i,1} \end{array}$$

(31) $$\begin{array}{cc} {\dot{\hat{\theta }}}_{i,1}= & -2\zeta_{i,1}\hat{\theta }-\eta_{i,1}{\tilde{z}}_{i,1}\rho_{i,1}\Xi_{i,1}\left({\underline{\hat{x}}}_{i,1}\right) \end{array}$$

where the designed parameters $a_{i,1}>0,b_{i,1}>0,g_{i,1}>0$, and $\varsigma_{i,1}>0$.

Combine (25)–(31), and one obtains

(32) $$\begin{array}{cc} {\dot{V}}_{i,1}\leq & -a_{i,1}\pi_{i,1}^{2}-\frac{b_{i,1}\pi_{i,1}^{2p}}{{\left(\epsilon_{i,1}^{2}-\pi_{i,1}^{2}\right)}^{p}}+\frac{2g_{i,1}}{\mu_{i,1}}{\tilde{\nu }}_{i,1}{\hat{\nu }}_{i,1}+\frac{m_{i,1}^{2}}{2}\\ + & \frac{{\bar{\Phi }}_{i,1}}{2}+\frac{\varsigma_{i,1}^{2}}{\eta_{i,1}}{\tilde{\theta }}_{i,1}{\hat{\theta }}_{i,1}+{\tilde{\theta }}_{i,1}{\tilde{z}}_{i,1}\rho_{i,1}\Xi_{i,1}\left({\underline{\hat{x}}}_{i,1}\right) \end{array}$$

*step m:* the m-order barrier Lyapunov function $V_{i,m}$ is as follows:

(33) $$\begin{array}{c} V_{i,m}=\frac{1}{2}log\frac{\epsilon_{i,m}^{2}}{\epsilon_{i,m}^{2}-\pi_{i,m}^{2}}+\frac{1}{2\mu_{i,m}}{\tilde{\nu }}_{i,m}^{2}+\frac{1}{2\eta_{i,m}}{\tilde{\theta }}_{i,m}^{2}+V_{i,m-1} \end{array}$$

where $|\pi_{i,m}|<\epsilon_{i,m}$, $\epsilon_{i,m}$ and $\eta_{i,m}$ are positive constants, and ${\tilde{\nu }}_{i,m}=\nu_{i,m}-{\hat{\nu }}_{i,m}$.

Based on (11), (12), and (24), one yields

(34) $$\begin{array}{cc} {\dot{\pi }}_{i,m}= & {\dot{\hat{x}}}_{i,m}-{\dot{\alpha }}_{i,m-1}\\ = & {\hat{x}}_{i,m+1}+{\hat{f}}_{i,m}\left({\underline{\hat{x}}}_{i,m}|{\hat{\theta }}_{i,m}\right)+l_{i,m}e_{i,1}+{\hat{d}}_{i,m}-{\dot{\alpha }}_{i,m-1}\\ = & {\hat{x}}_{i,m+1}+{\hat{f}}_{i,m}\left({\underline{\hat{x}}}_{i,m}|{\hat{\theta }}_{i,m}\right)+l_{i,m}e_{i,1}+{\hat{d}}_{i,m}-{\dot{\chi }}_{i,(m-1)2}-\varphi_{i,m-1} \end{array}$$

where $\varphi_{i,m-1}={\dot{\alpha }}_{i,m-1}-{\dot{\chi }}_{i,(m-1)2}$.

Similar to Step 1. By utilizing the RBF NNs, one obtains

(35) $$\begin{array}{cc} F_{i,m}\left({ℜ}_{i,m}\right)= & {\hat{f}}_{i,m}\left({\underline{\hat{x}}}_{i,m}|{\hat{\theta }}_{i,m}\right)+\pi_{i,m+1}+{\hat{d}}_{i,m}+e_{i,m+1}-\chi_{i,(m-1)2}\\ = & \varpi_{i,m}^{T}\Psi_{i,m}\left({ℜ}_{i,m}\right)+\Phi_{i,m}\left({ℜ}_{i,m}\right) \end{array}$$

where ${ℜ}_{i,m}={\left[{\underline{\hat{x}}}_{i,m},{\underline{\hat{x}}}_{j},{\hat{d}}_{i},{\hat{d}}_{j},{\hat{\theta }}_{i},{\bar{y}}_{0}\right]}^{T}$ with ${\bar{y}}_{0}={\left[y_{0},{\dot{y}}_{0},\ldots ,y_{0}^{m}\right]}^{T}$. Considering Young’s inequality, one obtains

(36) $$\begin{array}{cc} \varpi_{i,m}^{T}\Psi_{i,m}\left({ℜ}_{i,m}\right){\bar{\pi }}_{i,m}\leq & \frac{{\bar{\pi }}_{i,m}^{2}\nu_{i,m}\Psi_{i,m}^{T}\Psi_{i,m}}{2m_{i,m}^{2}}+\frac{m_{i,m}^{2}}{2}\\ \Phi_{i,m}{\bar{\pi }}_{i,m}\leq & \frac{{\bar{\pi }}_{i,m}^{2}}{2}+\frac{{\bar{\Phi }}_{i,m}^{2}}{2}\\ l_{i,m}e_{i,1}{\bar{\pi }}_{i,m}\leq & \frac{1}{2}{\bar{\pi }}_{i,m}^{2}+\frac{1}{2}{\bar{l}}_{i}e_{i}^{T}e_{i}\\ -\varphi_{i,m-1}{\bar{\pi }}_{i,m}\leq & \frac{1}{2}\varphi_{i,m-1}^{2}+\frac{1}{2}\bar{\pi }{i,m}^{2} \end{array}$$

Then $\alpha_{i,m}$, $\nu_{i,m}$ and $\theta_{i,m}$ are built as

(37) $$\begin{array}{cc} \alpha_{i,m}= & -a_{i,m}\pi_{i,m}-\frac{b_{i,m}\pi_{i,m}^{2p-1}}{{\left(\epsilon_{i,m}^{2}-\pi_{i,m}^{2}\right)}^{p-1}}-\frac{3{\bar{\pi }}_{i,m}^{2}}{2}-\frac{{\bar{\pi }}_{i,m}\nu_{i,m}\Psi_{i,m}^{T}\Psi_{i,m}}{2m_{i,m}^{2}} \end{array}$$

(38) $$\begin{array}{cc} {\dot{\hat{\nu }}}_{i,m}= & \frac{{\bar{\pi }}_{i,m}^{2}\Psi_{i,m}^{T}\Psi_{i,m}\mu_{i,m}}{2m_{i,m}^{2}}-2g_{i,m}{\hat{\nu }}_{i,m} \end{array}$$

(39) $$\begin{array}{cc} {\dot{\hat{\theta }}}_{i,m}= & -2\zeta_{i,m}{\hat{\theta }}_{i,m}-\eta_{i,m}{\tilde{z}}_{i,m}\rho_{i,m}\Xi_{i,m}\left({\underline{\hat{x}}}_{i,m}\right) \end{array}$$

Combining (33)–(39), we obtain the differentiation of $V_{i,m}$ as follows:

(40) $$\begin{array}{cc} {\dot{V}}_{i,m}\leq & -\sum_{k=1}^{m}\frac{a_{i,k}\pi_{i,k}^{2}}{\epsilon_{i,k}^{2}-\pi_{i,k}^{2}}-\sum_{k=1}^{m}\frac{b_{i,k}\pi_{i,k}^{2p}}{{\left(\epsilon_{i,k}^{2}-\pi_{i,k}^{2}\right)}^{p}}\\ + & \sum_{k=1}^{m}\frac{2g_{i,k}}{\mu_{i,k}}{\tilde{\nu }}_{i,k}{\hat{\nu }}_{i,k}+\sum_{k=1}^{m}\frac{m_{i,k}^{2}}{2}+\sum_{k=1}^{m}\frac{{\bar{\Phi }}_{i,k}^{2}}{2}\\ + & \sum_{k=1}^{m}\frac{\varsigma_{i,k}^{2}}{\eta_{i,k}}{\tilde{\theta }}_{i,k}{\hat{\theta }}_{i,k}+\sum_{k=1}^{m}{\tilde{\theta }}_{i,1}{\tilde{z}}_{i,k}\rho_{i,k}\Xi_{i,k}\left({\underline{\hat{x}}}_{i,k}\right)\\ + & \frac{1}{2}\sum_{k=2}^{m}\varphi_{i,k-1}^{2}+\frac{1}{2}m{\bar{l}}_{i}e_{i}^{T}e_{i} \end{array}$$

*step $n_{i}$:* to reduce the frequency of triggering and save resources, the event-triggered technique will be proposed. The adaptive controller can be defined as follows:

(41) $$\begin{array}{c} \beta_{i}\left(t\right)=-\alpha_{i,n_{i}}\tanh\left(\frac{{\bar{\pi }}_{i,n_{i}}\alpha_{i,n_{i}}}{\varrho_{i,1}}\right)\left(1+\xi_{i}\right)-\frac{\left(1+\xi_{i}\right){\bar{\pi }}_{i,n_{i}}}{2(1-\xi_{i})} \end{array}$$

(42) $$\begin{array}{c} \left\{\begin{array}{cc} t_{i,m+1}= & \inf\{t\in R||\phi_{i}\left(t\right)|\geq q_{i}+\xi_{i}|u_{i}\left(t\right)|\}\\ u_{i}\left(t\right)= & \beta_{i}\left(t_{i,m}\right),\forall t\in \left[t_{i,m},t_{i,m+1}\right) \end{array}\right. \end{array}$$

where $q_{i}>0,\varrho_{i}>0,0<\xi_{i}<1$ and $\phi_{i}\left(t\right)=\beta_{i}\left(t\right)-\alpha_{i}\left(t\right)$.

**Remark** **1.** *Based on (41) and (42), if $|\phi_{i}\left(t\right)|\geq q_{i}+\xi_{i}\left|u_{i}\left(t\right)\right|$, the controller updates and keeps the new constant unless next triggering, which means that as long as $\phi_{i}\left(t\right)$ exceeds the threshold signal $q_{i}+\xi_{i}\left|u_{i}\left(t\right)\right|$, the control input will update. $u_{i}\left(t\right)=\beta_{i}\left(t_{i,m+1}\right)$ keeps unless the next triggering.*

It will be obtained that $u_{i}\left(t\right)=\frac{\beta_{i}\left(t\right)-q_{i}\psi_{i,1}\left(t\right)}{1+\xi_{i}\psi_{i,2}\left(t\right)}$ with $|\psi_{i,1}\left(t\right)|\leq 1$ and $|\psi_{i,2}\left(t\right)|\leq 1$. Hence, one obtains

(43) $$\begin{array}{cc} {\bar{\pi }}_{i,n_{i}}u_{i}= & -{\bar{\pi }}_{i,n_{i}}(\frac{1+\xi_{i}}{1+\xi_{i}\psi_{i,2}\left(t\right)}\left(\alpha_{i,n_{i}}\tanh\left(\frac{{\bar{\pi }}_{i,n_{i}}\alpha_{i,n_{i}}}{\varrho_{i,1}}\right)\right)\\ + & \frac{\left(1+\xi_{i}\right){\bar{\pi }}_{i,n_{i}}}{2\left(1+\xi_{i}\psi_{i,2}\left(t\right)\right){\left(1-\xi_{i}\right)}^{2}}+\frac{q_{i}\psi_{i,1}\left(t\right)}{1+\xi_{i}\psi_{i,2}\left(t\right)}) \end{array}$$

According to [33], for ∀o∈R and $\lambda >0,-o\tanh(\frac{o}{\lambda })\leq 0$ and $0\leq |o|-o\tanh(\frac{o}{\lambda })\leq 0.2785\lambda$ are obtained. Hence, one yields

(44) $$\begin{array}{cc} {\bar{\pi }}_{i,n_{i}}u_{i}\leq & |{\bar{\pi }}_{i,n_{i}}\alpha_{i,n_{i}}|-{\bar{\pi }}_{i,n_{i}}\alpha_{i,n_{i}}\tanh\left(\frac{{\bar{\pi }}_{i,n_{i}}\alpha_{i,n_{i}}}{\varrho_{i,1}}\right)\\ - & |{\bar{\pi }}_{i,n_{i}}\alpha_{i,n_{i}}\left|+\right|\frac{q_{i}{\bar{\pi }}_{i,n_{i}}}{1-\xi_{i}}|-\frac{{\bar{\pi }}_{i,n_{i}}^{2}}{2{\left(1-\xi_{i}\right)}^{2}}\\ \leq & {\bar{\pi }}_{i,n_{i}}\alpha_{i,n_{i}}+\frac{q_{i}^{2}}{2}+0.2785\varrho_{i,1} \end{array}$$

The barrier Lyapunov function $V_{i,n_{i}}$ will be chosen as follows:

(45) $$\begin{array}{cc} V_{i,n_{i}}= & \frac{1}{2}log\frac{\epsilon_{i,n_{i}}^{2}}{\epsilon_{i,n_{i}}^{2}-\pi_{i,n_{i}}^{2}}+\frac{1}{2\mu_{i,n_{i}}}{\tilde{\nu }}_{i,n_{i}}^{2}+\frac{1}{2\eta_{i,n_{i}}}{\tilde{\theta }}_{i,n_{i}}^{2}\\ + & V_{i,n_{i}-1} \end{array}$$

Similar to Step 1, use RBF NNs to deal with uncertain terms, as follows:

(46) $$\begin{array}{cc} F_{i,n_{i}}\left({ℜ}_{i,n_{i}}\right)= & {\hat{f}}_{i,n_{i}}\left({\underline{\hat{x}}}_{i,n_{i}}|{\hat{\theta }}_{i,n_{i}}\right)+{\hat{d}}_{i,n_{i}}-\chi_{i,(n_{i}-1)2}\\ = & \varpi_{i,n_{i}}^{T}\Psi_{i,n_{i}}\left({ℜ}_{i,n_{i}}\right)+\Phi_{i,n_{i}}\left({ℜ}_{i,n_{i}}\right) \end{array}$$

Then, the $\alpha_{i,n_{i}}$, $\nu_{i,n_{i}}$ and $\theta_{i,n_{i}}$ are constructed as follows:

(47) $$\begin{array}{cc} \alpha_{i,n_{i}}= & -a_{i,n_{i}}\pi_{i,n_{i}}-\frac{b_{i,n_{i}}\pi_{i,n_{i}}^{2p-1}}{{\left(\epsilon_{i,n_{i}}^{2}-\pi_{i,n_{i}}^{2}\right)}^{p-1}}-\frac{3{\bar{\pi }}_{i,n_{i}}^{2}}{2}-\frac{{\bar{\pi }}_{i,n_{i}}\nu_{i,n_{i}}\Psi_{i,n_{i}}^{T}\Psi_{i,n_{i}}}{2m_{i,n_{i}}^{2}} \end{array}$$

(48) $$\begin{array}{cc} {\dot{\hat{\nu }}}_{i,n_{i}}= & \frac{{\bar{\pi }}_{i,n_{i}}^{2}\Psi_{i,n_{i}}^{T}\Psi_{i,n_{i}}\mu_{i,n_{i}}}{2m_{i,n_{i}}^{2}}-2g_{i,n_{i}}{\hat{\nu }}_{i,n_{i}} \end{array}$$

(49) $$\begin{array}{cc} {\dot{\hat{\theta }}}_{i,n_{i}}= & -2\zeta_{i,n_{i}}{\hat{\theta }}_{i,n_{i}}-\eta_{i,n_{i}}{\tilde{z}}_{i,n_{i}}\rho_{i,n_{i}}\Xi_{i,n_{i}}\left({\underline{\hat{x}}}_{i,n_{i}}\right) \end{array}$$

Combining (43)–(49), one obtains

(50) $$\begin{array}{cc} {\dot{V}}_{i,n_{i}}\leq & -\sum_{k=1}^{n_{i}}\frac{a_{i,k}\pi_{i,k}^{2}}{\epsilon_{i,k}^{2}-\pi_{i,k}^{2}}-\sum_{k=1}^{n_{i}}\frac{b_{i,k}\pi_{i,k}^{2p}}{{\left(\epsilon_{i,k}^{2}-\pi_{i,k}^{2}\right)}^{p}}\\ + & \sum_{k=1}^{n_{i}}\frac{2g_{i,k}}{\mu_{i,k}}{\tilde{\nu }}_{i,k}{\hat{\nu }}_{i,k}+\sum_{k=1}^{n_{i}}\frac{m_{i,k}^{2}}{2}+\sum_{k=1}^{n_{i}}\frac{{\bar{\Phi }}_{i,k}}{2}\\ + & \sum_{k=1}^{n_{i}}\frac{\varsigma_{i,k}^{2}}{\eta_{i,k}}{\tilde{\theta }}_{i,k}{\hat{\theta }}_{i,k}+\sum_{k=1}^{n_{i}}{\tilde{\theta }}_{i,1}{\tilde{z}}_{i,k}\rho_{i,k}\Xi_{i,k}\left({\underline{\hat{x}}}_{i,k}\right)\\ + & \frac{1}{2}\sum_{k=2}^{n_{i}}\varphi_{i,k-1}^{2}+\frac{1}{2}n_{i}{\bar{l}}_{i}e_{i}^{T}e_{i}+\frac{q_{i}^{2}}{2}+0.2785\varrho_{i,1} \end{array}$$

## 5. Stability Analysis

This section concentrates on proving that the Lyapunov function converges based on the above discussion and derivation of formulas.

**Theorem** **1.** *For nonlinearMASs (1), discuss the virtual and actual controllers (29), (37), and (47) as well as adaptive laws (30), (31), (38), (39), (48), and (49) under Assumptions 1 and 2. Meanwhile, consider the state observer (12), disturbance observer (14), and event-triggered mechanism (41). Ultimately, all signals are bounded and the consensus error between the agents is in a small region.*

Select a total barrier Lyapunov function as

(51) $$\begin{array}{c} V=\sum_{i=1}^{N}\sum_{k=1}^{n_{i}}V_{i,k}+V_{e} \end{array}$$

Based on Lemmas 2 and 3 [22,28] and Young’s inequality, we obtain

(52) $$\begin{array}{cc} \log\frac{\epsilon_{i,k}^{2}}{\epsilon_{i,k}^{2}-\pi_{i,k}^{2}}\leq & \frac{\epsilon_{i,k}^{2}}{\epsilon_{i,k}^{2}-\pi_{i,k}^{2}}\\ \sum_{k=1}^{n_{i}}\frac{2g_{i,k}}{\mu_{i,k}}{\tilde{\nu }}_{i,k}{\hat{\nu }}_{i,k}\leq & \sum_{k=1}^{n_{i}}\frac{g_{i,k}}{\mu_{i,k}}\nu_{i,k}^{2}-\sum_{k=1}^{n_{i}}\frac{g_{i,k}}{\mu_{i,k}}{\tilde{\nu }}_{i,k}^{2}\\ \sum_{k=1}^{n_{i}}\frac{2\varsigma_{i,k}}{\eta_{i,k}}{\tilde{\theta }}_{i,k}{\hat{\theta }}_{i,k}\leq & \sum_{k=1}^{n_{i}}\frac{\varsigma_{i,k}}{\eta_{i,k}}\theta_{i,k}^{2}+\sum_{k=1}^{n_{i}}\frac{\varsigma_{i,k}}{\eta_{i,k}}{\tilde{\theta }}_{i,k}^{2}\\ {\left(\sum_{k=1}^{n_{i}}\frac{{\tilde{\nu }}_{i,k}^{2}}{2}\right)}^{p}\leq & \sum_{k=1}^{n_{i}}\frac{{\tilde{\nu }}_{i,k}^{2}}{2}+{\left(1-p\right)}^{\frac{p}{1-p}}\\ {\left(\sum_{k=1}^{n_{i}}\frac{{\tilde{\theta }}_{i,k}^{2}}{2}\right)}^{p}\leq & \sum_{k=1}^{n_{i}}\frac{{\tilde{\theta }}_{i,k}^{2}}{2}+{\left(1-p\right)}^{\frac{p}{1-p}} \end{array}$$

Combining (22), (50), (51), and (52), one obtains

(53) $$\begin{array}{cc} \dot{V}\leq & -\gamma_{1}\left(\frac{1}{2}\sum_{i=1}^{N}\sum_{k=1}^{n_{i}}\log\frac{a_{i,k}\pi_{i,k}^{2}}{\epsilon_{i,k}^{2}-\pi_{i,k}^{2}}\right)-\gamma_{2}(\frac{1}{2}\sum_{i=1}^{N}\sum_{k=1}^{n_{i}}\log\frac{b_{i,k}\pi_{i,k}^{2}}{\epsilon_{i,k}^{2}}\\ - & \pi_{i,k}^{2}{)}^{p}-\gamma_{3}\sum_{i=1}^{N}∥e_{i}{∥}^{2}-\gamma_{3}\sum_{i=1}^{N}{∥e_{i}∥}^{2p}-\gamma_{4}\sum_{i=1}^{N}\sum_{k=1}^{n_{i}}\frac{{\tilde{z}}_{i,k}^{2}}{2}\\ - & \gamma_{4}{\left(\sum_{i=1}^{N}\sum_{k=1}^{n_{i}}\frac{{\tilde{z}}_{i,k}}{2}\right)}^{2p}-\gamma_{5}\sum_{i=1}^{N}\sum_{k=1}^{n_{i}}\frac{1}{2\mu_{i,k}}{\tilde{\nu }}_{i,k}^{2}-\gamma_{5}{\left(\sum_{i=1}^{N}\sum_{k=1}^{n_{i}}\frac{1}{2\mu_{i,k}}{\tilde{\nu }}_{i,k}^{2}\right)}^{p}\\ - & \gamma_{6}\sum_{i=1}^{N}\sum_{k=1}^{n_{i}}\frac{1}{2\eta_{i,k}}{\tilde{\theta }}_{i,k}^{2}-\gamma_{6}{\left(\sum_{i=1}^{N}\sum_{k=1}^{n_{i}}\frac{1}{2\eta_{i,k}}{\tilde{\theta }}_{i,k}^{2}\right)}^{p}+\sum_{i=1}^{N}R_{i} \end{array}$$

with

(54) $$\begin{array}{cc} R_{i}= & \left(\frac{\rho_{i}^{2}}{2}+2\right){\bar{\bar{\Delta }}}_{i}+\frac{1}{2}n_{i}\kappa_{2}^{2}+\sum_{k=1}^{n_{i}}\left(\frac{m_{i,k}^{2}}{2}+\frac{{\bar{\Phi }}_{i,k}^{2}}{2}\right)+\frac{1}{2}\sum_{k=2}^{n_{i}}\varphi_{i,k-1}^{2}+\frac{q_{i}^{2}}{2}\\ + & \gamma_{3}{∥e_{i}∥}^{2p}+\gamma_{4}{\left(\sum_{k=1}^{n_{i}}\frac{{\tilde{z}}_{i,k}}{2}\right)}^{2p}+\sum_{k=1}^{n_{i}}\frac{g_{i,k}}{\mu_{i,k}}\nu_{i,k}^{2}+\sum_{k=1}^{n_{i}}\frac{\varsigma_{i,k}}{\eta_{i,k}}\theta_{i,k}^{2}\\ + & 2{\left(1-p\right)}^{\frac{p}{1-p}} \end{array}$$

where $\gamma_{1}=2\min\left\{a_{i,1},\ldots ,a_{i,n_{i}}\right\}$, $\gamma_{2}=2^{p}\min\left\{b_{i,1},\ldots ,b_{i,n_{i}}\right\}$, $\gamma_{3}=\min\left\{Q_{i}\right\}$ with $Q_{i}=\lambda_{min}\left(N_{i}\right)-\frac{1}{2}\left(n_{i}-1\right){\bar{\rho }}_{i}^{2}-\frac{1}{2}n_{i}{\bar{\rho }}_{i}^{4}-n_{i}-\left(n_{i}+n_{i}{\bar{\rho }}_{i}\right)∥M_{i}{∥}_{F}-\frac{1}{2}n_{i}{\bar{l}}_{i}-{∥M_{i}∥}_{F}^{2}>0$, $\gamma_{4}=\min\{2-\rho_{i,m}+∥M_{i}{∥}_{F}^{2},\frac{3}{2}-\rho_{i,n_{i}}+∥M_{i}{∥}_{F}^{2}\}$, $\gamma_{5}=min\left\{4g_{i,1},\ldots ,4g_{i,n_{i}}\right\}$ and $\gamma_{6}=min\left\{4\varsigma_{i,1},\ldots ,4\varsigma_{i,n_{i}}\right\}$.

Then, for two positive unknown constants $\Omega_{1},\Omega_{2}$ such that $|e_{i}|\leq \Omega_{1}$ and $|{\tilde{z}}_{i}|\leq \Omega_{2}$, respectively.

Finally, one obtains

(55) $$\begin{array}{cc} \dot{V}\leq & -\gamma \sum_{i=1}^{N}V_{i,n_{i}}-\sigma {\left(\sum_{i=1}^{N}V_{i,n_{i}}\right)}^{p}+R\\ = & -\gamma V-\sigma V^{p}+R \end{array}$$

where p∈(0,1), $\gamma =\min\{\gamma_{1},\gamma_{3},\ldots ,\gamma_{N}\}$, $\sigma =\min\{\gamma_{2},\gamma_{3},\ldots ,\gamma_{N}\}$, and $R=\sum_{i=1}^{N}R_{i}$.

Based on Lemma 1, the settling time is

(56) $$\begin{array}{cc} T_{e}\leq \max\{t_{0}+ & \frac{1}{\Lambda \gamma (1-p)}\ln\frac{\Lambda \gamma V^{1-p}\left(t_{0}\right)+\sigma }{\sigma },\\ t_{0} & +\frac{1}{\gamma (1-p)}\ln\frac{\gamma V^{1-p}\left(t_{0}\right)+\Lambda \sigma }{\Lambda \sigma }\} \end{array}$$

**Remark** **2.** *From Lemma 1 and (56), it can be shown that the error signal is bounded. In addition, we also have that ${\hat{\nu }}_{i,1},\ldots ,{\hat{\nu }}_{i,n_{i}},{\hat{\theta }}_{i,1},\ldots ,{\hat{\theta }}_{i,n_{i}},\alpha_{i,1},\ldots ,\alpha_{i,n_{i}}$ are bounded.*

**Remark** **3.** *Based on [1], it is common to occur that the tracking error satisfies $∥y_{i,1}-y_{0}∥\leq \frac{|\pi |}{\lambda_{max}\left(L+B\right)}$ and ∣π∣ is the synchronization error. Meanwhile, all system state signals $|x_{i}|\leq \epsilon_{i}$, which satisfy the full-state constraint.*

**Remark** **4.** *Owing to the waste of communication resources, the adaptive controller (41) can be designed to reduce the amount of triggering. From (42), we can adjust the designed parameter to achieve optimal control performance based on limited triggering. We have $|{\dot{\beta }}_{i}|\leq {\bar{\beta }}_{i}$ with ${\bar{\beta }}_{i}>0$. Based on (41) and (42), if $t\in (t_{i,m},t_{i,m+1})$, $\frac{d|\phi_{i}\left(t\right)|}{dt}\leq |{\dot{\beta }}_{i}|$ can be established. Additionally, $\lim_{t\to t_{i,m+1}}\phi_{i}\left(t\right)=q_{i}+\xi_{i}\left|u_{i}\left(t\right)\right|$, and $\phi_{i}\left(t\right)=0$ such that the interval of the triggered events has $t_{i}^{★}\geq \frac{q_{i}+\xi_{i}\left|u_{i}\left(t\right)\right|}{{\bar{\beta }}_{i}}$, so the Zeno behavior can be avoided.*

**Remark** **5.** *When the virtual control methods are proposed, the multiple derivations of virtual controllers will cause the issues of ‘Complexity explosion’, which motivates the introduction of Tracking Differentiator (TD) (10) and (11). TD is also able to avoid the chattering by using sat(•). Seeing the traditional first-order filter [34], the filter can easily cause instability because it can be influenced by constant γ. So it does not have better precision than TD.*

## 6. Simulation Results

**Figure 1:**
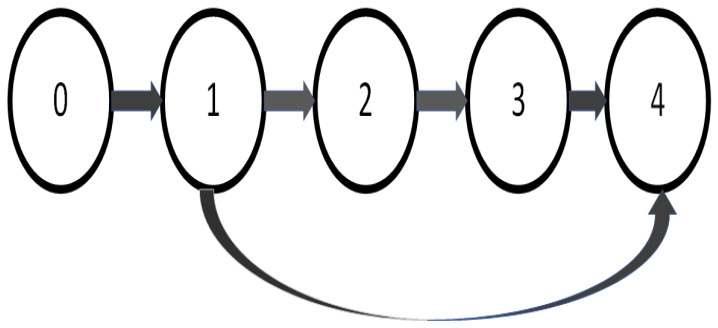
Communication topology of the agents.

In this part, to confirm that the consensus control technique is valid, a simulated example is given. Firstly, the reference signal is $y_{0}=0.5\sin\left(0.4t\right)$. Furthermore, the communication digraph is displayed in Figure 1. Based on Figure 1, one obtains

$$A^{*}=\left[\begin{array}{cccc} 0 & 0 & 0 & 0\\ 1 & 0 & 0 & 0\\ 0 & 1 & 0 & 0\\ 1 & 0 & 1 & 0 \end{array}\right],L^{*}=\left[\begin{array}{cccc} 0 & 0 & 0 & 0\\ -1 & 1 & 0 & 0\\ 0 & -1 & 1 & 0\\ -1 & 0 & -1 & 1 \end{array}\right]$$

Consider the four followers’ system model

(57) $$\begin{array}{c} \left\{\begin{array}{c} {\dot{x}}_{i,1}=x_{i,2}+f_{i,1}\left({\underline{x}}_{i,1}\right)+d_{i,1}\left(t\right)\\ {\dot{x}}_{i,2}=u_{i}+f_{i,2}\left({\underline{x}}_{i,2}\right)+d_{i,2}\left(t\right),i=1,2,3,4\\ y_{i}=x_{i,1} \end{array}\right. \end{array}$$

where the unknown functions $f_{i,1}=x_{i,1}^{2}\sin\left(x_{i,2}\right),f_{i,2}=\sin\left(x_{i,1}x_{i,2}\right)$, and the external disturbance is given by $d_{i,1}\left(t\right)=x_{i,1}\sin\left(x_{i,2}\right)\cos\left(t\right),d_{i,2}=1+x_{i,1}x_{i,2}\sin{\left(t\right)}^{2}$. The initial conditions of the system can be designed as ${\underline{x}}_{1}={\left[0.1,0.2\right]}^{T},{\underline{x}}_{2}={\left[0.1,0.1\right]}^{T},{\underline{x}}_{3}={\left[0.1,0.2\right]}^{T},{\underline{x}}_{i,1}={\left[0.1,0.1\right]}^{T}$, ${\underline{\hat{x}}}_{1}={\left[0.2,0.1\right]}^{T},{\underline{\hat{x}}}_{2}={\left[0,-0.1\right]}^{T},{\underline{\hat{x}}}_{3}={\left[0.1,0\right]}^{T},{\underline{\hat{x}}}_{i,1}={\left[0.1,0.2\right]}^{T}$.

Then, the state observer is designed as follows:

(58) $$\begin{array}{c} \left\{\begin{array}{c} {\dot{\hat{x}}}_{i,1}={\hat{x}}_{i,2}+{\hat{f}}_{i,1}\left({\underline{\hat{x}}}_{i,1}|{\hat{\theta }}_{i,1}\right)+l_{i,1}\left(y_{i}-{\hat{x}}_{i,1}\right)+{\hat{d}}_{i,1}\left(t\right)\\ {\dot{\hat{x}}}_{i,2}=u_{i}+{\hat{f}}_{i,2}\left({\underline{\hat{x}}}_{i,2}|{\hat{\theta }}_{i,2}\right)+l_{i,2}\left(y_{i}-{\hat{x}}_{i,1}\right)+{\hat{d}}_{i,2}\left(t\right)\\ {\hat{y}}_{i}={\hat{x}}_{i,1} \end{array}\right. \end{array}$$

in which $l_{i,1}=65,$ $l_{i,2}=30$. Furthermore, the other main parameters are given: $p=\frac{4}{5},$ $a_{i,1}=18,$ $a_{i,2}=35,$ $b_{i,1}=12,$ $b_{i,2}=25,$ $c_{i}=15,$ $\epsilon_{i,1}=1,$ $\epsilon_{i,2}=3,$ $\mu_{i,1}=0.5,$ $\mu_{i,2}=0.5,$ $g_{i,1}=0.5,$ $g_{i,2}=0.5,$ $m_{i,1}=0.5,$ $m_{i,2}=0.5,$ $\zeta_{i,1}=0.1,$ $\zeta_{i,2}=0.1,$ $\eta_{i,1}=0.2,$ $\eta_{i,2}=0.2,$ $\rho_{i,1}=30$, and $\rho_{i,2}=20$.

**Figure 2:**
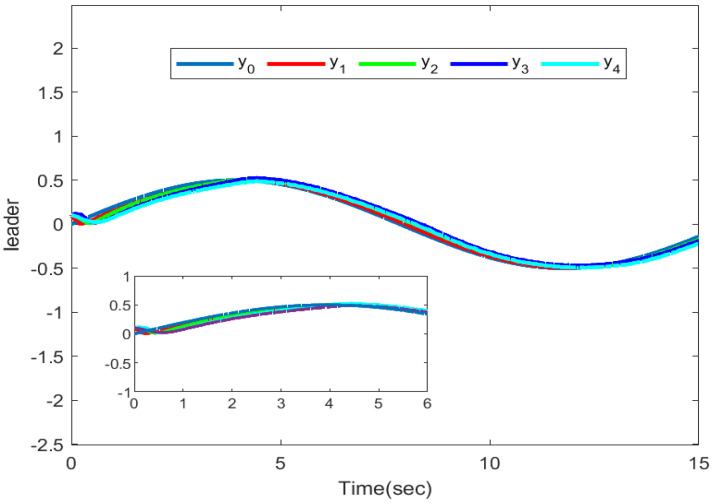
Tracking curves.

**Figure 3:**
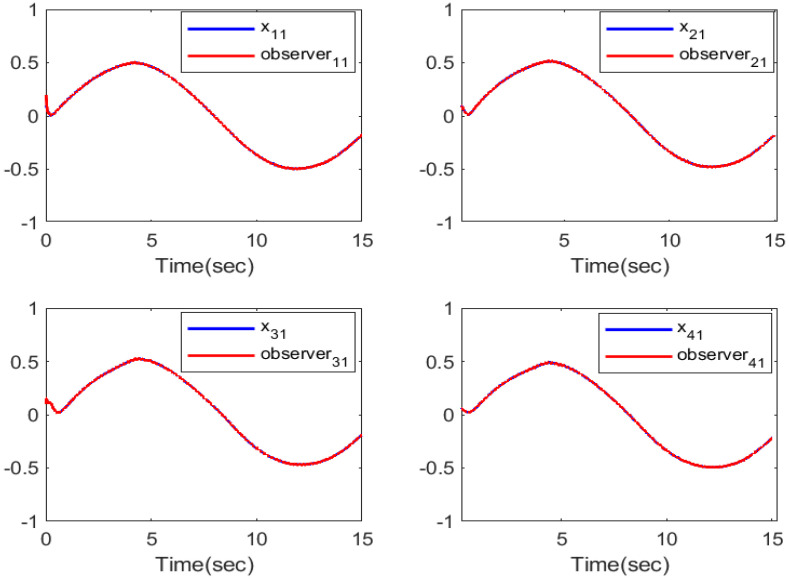
The first-order state observer and system states.

**Figure 4:**
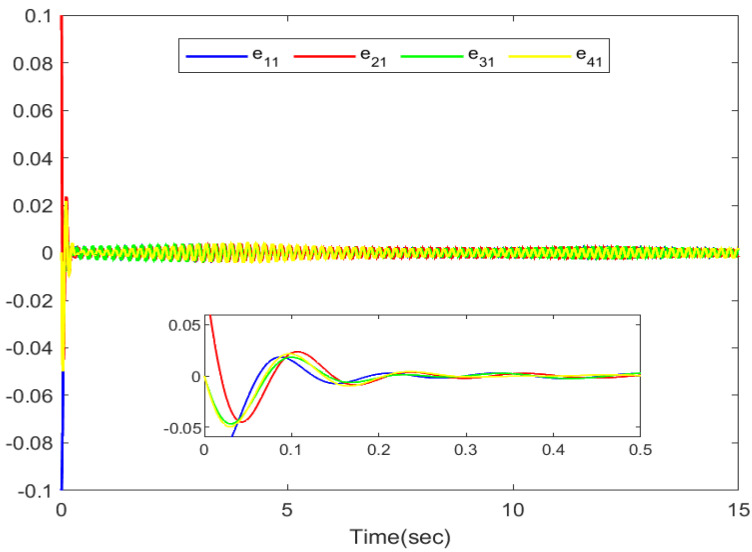
The error of the first-order state observers.

**Figure 5:**
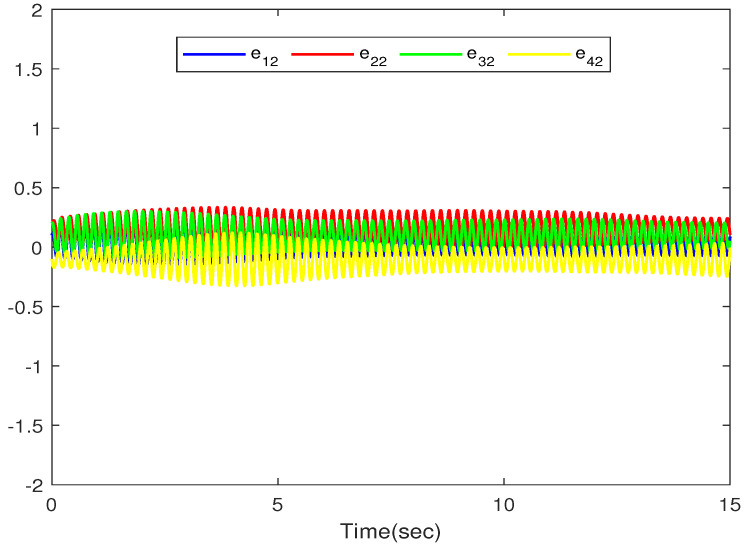
The error of the second-order state observers.

**Figure 6:**
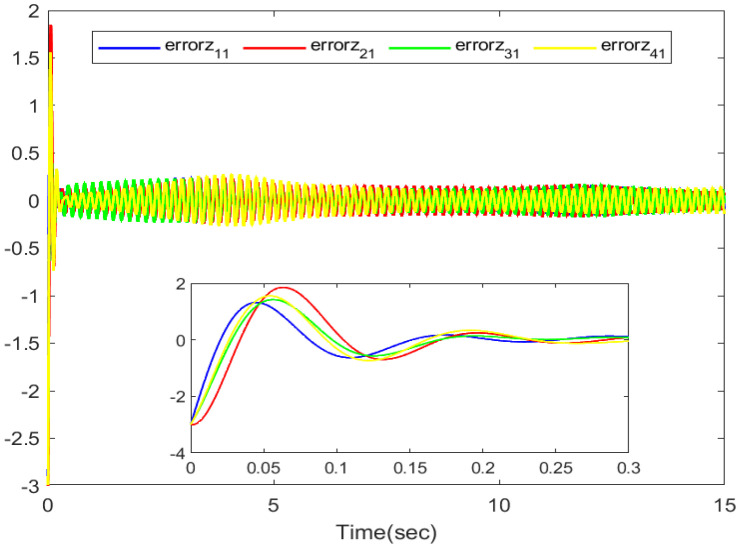
The error of the first-order disturbance observers.

**Figure 7:**
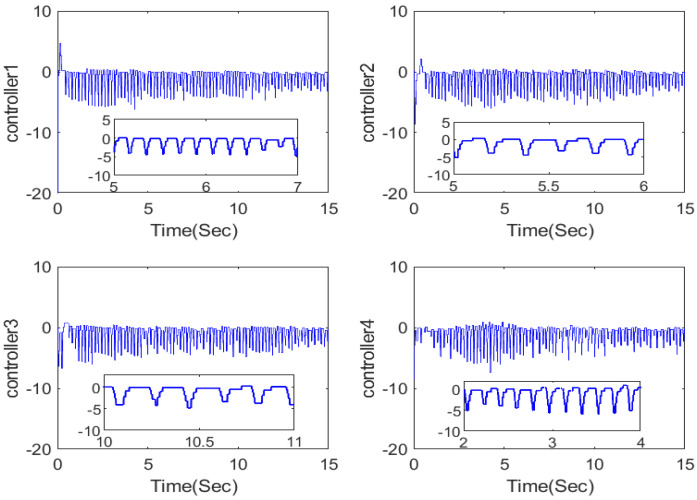
Adaptive event-triggered controller.

**Figure 8:**
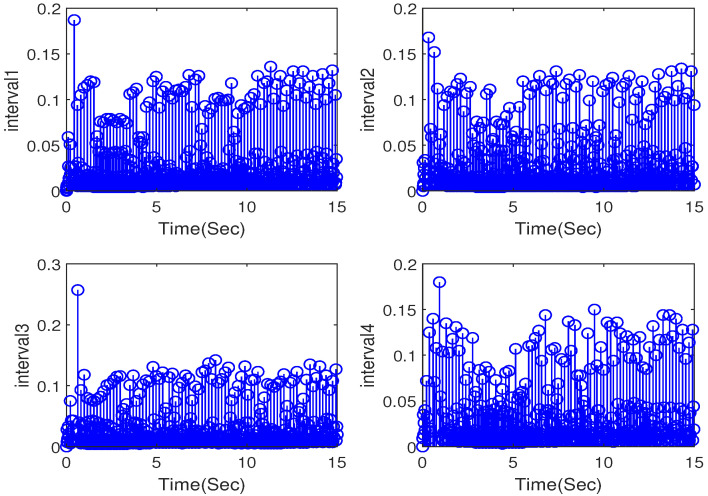
The interevent times of controller.

The simulation outputs are displayed in Figure 2, Figure 3, Figure 4, Figure 5, Figure 6, Figure 7 and Figure 8. Figure 2 exhibits the tracking consensus cases. Figure 3 illustrates the state observers and system states. Figure 4, Figure 5 and Figure 6 illustrate the errors of the state observers and disturbance observers. The adaptive event-triggered controller and the frequency of triggering are shown in Figure 7 and Figure 8. The number of controllers triggering is 540, 548, 520, and 486, respectively.

## 7. Conclusions

This paper is mainly meant to design the fast finite-time event-triggered control scheme for a class of MASs with state restrictions. By utilizing the designed approach, the tracking consensus control can be finished at a relatively high speed compared with previous techniques. Meanwhile, the system resources would be saved in practical applications and the Zeno behavior would be avoided. Additionally, the state observer and the disturbance observer are proposed to deal with the unavailable states and disturbances, which enlarge the practicability of the method. Moreover, TD can address the ‘Complexity explosion’ effectively. Finally, the authors will focus on the input quantization and unidentified hysteresis in the future.

## Data Availability

Data is contained within the article.
